# Transjugular intrahepatic portosystemic shunt for Budd–Chiari syndrome with diffuse occlusion of hepatic veins

**DOI:** 10.1038/srep36380

**Published:** 2016-11-02

**Authors:** Fuliang He, Hongwei Zhao, Shan Dai, Yingfeng Wu, Lei Wang, Hongdong Huang, Zhendong Yue, Zhenhua Fan, Xiaoqun Dong, Fuquan Liu

**Affiliations:** 1 Department of Interventional Therapy, Beijing Shijitan Hospital, Capital Medical University, The 9th Affiliated Hospital of Peking University, P.R. 100038, China; 2 Department of Vascular Surgery, Beijing Xuanwu Hospital, Capital Medical University, P.R. 100053, China; 3 Division of Nephrology, Beijing Shijitan Hospital, Capital Medical University, The 9th Affiliated Hospital of Peking University, Beijing 100038, P.R. China; 4 Department of Gastroenterology, Stephenson Cancer Center, Department of Internal Medicine, College of Medicine, The University of Oklahoma Health Sciences Center, 73104, USA

## Abstract

Either acute or sub-acute Budd–Chiari syndrome (BCS) with diffuse occlusion of hepatic veins has a high mortality rate and remains challenging for clinical treatment. We aimed to evaluate the feasibility and safety of transjugular intrahepatic portosystemic shunt (TIPS) as a treatment for BCS with diffuse occlusion of hepatic veins. From January 2007 to December 2010, 100 patients were randomly recruited onto this study and 91 patients were treated with TIPS. 14 patients were defined as acute BCS group and 86 patients as sub-acute group. Patients with acute BCS had a significantly higher rate of jaundice whereas a lower rate of abdominal and chest varices, gastroesophageal variceal bleeding and refractory ascites than sub-acute group (P < 0.001). TIPS was technically successful in all 91 patients (12 in acute group). The portosystemic pressure gradient (PSG) was decreased to normal level, while total bilirubin (TBIL) and liver function were significantly improved. During follow-up period, the mortality rate of 91 patients who underwent TIPS was 6.59% (6/91), whereas 88.89% of 9 patients who didn’t receive TIPS procedure (2 in acute group). Collectively, TIPS is an effective and safe approach in treating BCS with diffuse occlusion of hepatic veins, which should be performed in time.

Budd–Chiari syndrome (BCS) is an uncommon hepatic disease resulting from hepatic venous obstruction at the level of hepatic vein (HV), inferior vena cava (IVC), or hepatic venules^1^. It occurs in 1 out of a million individuals in western countries and 10 out of a million persons in China^2^, characterized by the classical triad of abdominal pain, ascites, and liver enlargement. Diffuse occlusion of all the three levels of HVs often results in acute (suddenly complete HVs occlusion) and sub-acute BCS (rapidly progressive complete HVs occlusion), which has a mortality rate of over 90% remains challenging for surgeons, physicians and interventional radiologists^3^. Patients with acute and sub-acute BCS present with symptoms including digestive discomfort, abdominal distention, abdominal pain, severe jaundice, refractory ascites, hepatosplenomegaly, portal hypertension, gastrointestinal bleeding and even liver failure. The symptoms can be lethal without proper treatment^4^.

Unfortunately, no standard protocol of therapy has been established. The patients barely benefit from conservative treatment. Liver transplantation may be a choice, while nearly impossible to get a donor liver during a short period of time^5^. Thus it’s urgent to figure out an efficient solution for BCS with complete occlusion of all three hepatic veins.

From January 2007 and December 2010, 91 patients with acute and sub-acute BCS with diffuse occlusion of hepatic veins underwent transjugular intrahepatic portosystemic shunt (TIPS) in a single center, and a good clinical outcome was achieved. We compared the clinical features of patients in acute and sub-acute BCS group, and evaluated the feasibility and safety of TIPS as a promising avenue for these patients.

## Results

### Characteristics of study population

From January 2007 to December 2010, 377 patients with BCS were randomly recruited in Beijing Shijitan Hospital, and 100 patients (F/M: 66/34) were included in this study according to the inclusion and exclusion criteria. 14 patients (F/M: 8/4) were defined as acute BCS group, 86 patients (F/M: 58/28) as sub-acute BCS group. 91 patients (12 in acute BCS group and 79 in sub-acute group) underwent TIPS procedure. 9 patients (F/M: 5/4) (2 in acute group and 7 in sub-acute group) received conservative treatment (non-TIPS group) including liver protection therapy, HE prevention and supportive care.

**Figure 1:**
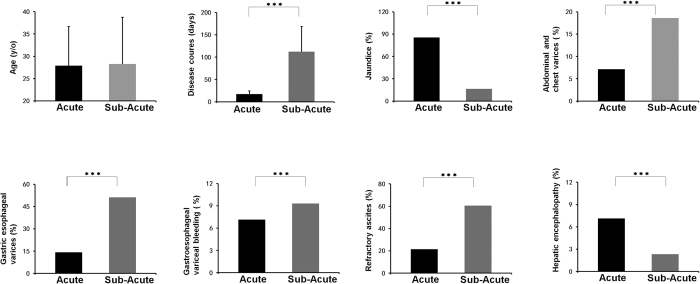
Clinical parameters of the patients. *P < 0.05; **P < 0.01; ***P < 0.001.

Preoperative clinical characteristics of the patients were listed in Fig. 1. There was no difference in average age between acute and sub-acute groups. The course of disease of acute BCS (17.14 ± 6.78 days) was significantly shorter than sub-acute group (112.40 ± 52.25 days) (P < 0.001). Patients with acute BCS had a significantly higher rate of jaundice (85.71%) than sub-acute group (16.28%) (P < 0.001). The incidence of abdominal and chest varices (7.14%), gastric esophageal varices (14.29%), gastroesophageal variceal bleeding (7.14%) and refractory ascites (21.43%) were all significantly lower in acute group than in sub-acute group (18.60%, 51.16%, and 60.47%, respectively) (P < 0.001). The rate of HE was significantly higher in acute BCS group (7.14%) than in sub-acute group (2.33%) (P < 0.001).

### TIPS procedure and efficacy

**Figure 2:**
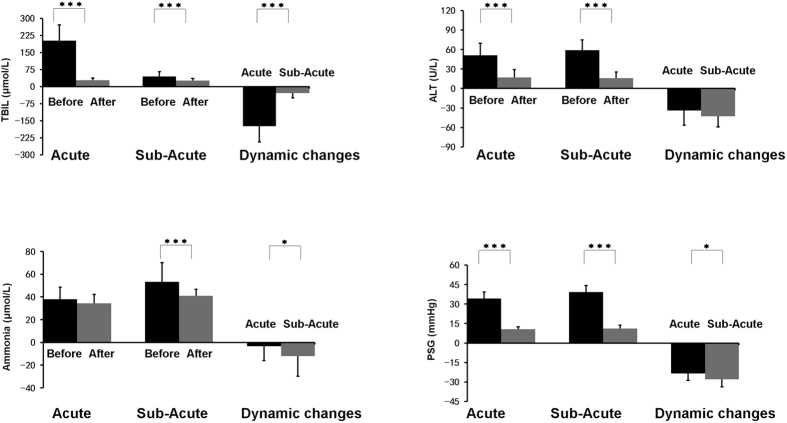
Laboratory and hemodynamic parameters before and after TIPS. *P < 0.05; **P < 0.01; ***P < 0.001.

TIPS was technically successful in all 91 patients (100.00%). A functional canal from portal vein to systemic vein was established. PSG of acute BCS was significantly decreased from 34.17 ± 5.06 to 10.66 ± 1.83 mmHg (P < 0.001), and PSG of sub-acute BCS was significantly decreased from 39.22 ± 4.90 to 11.15 ± 2.56 mmHg (P < 0.001) as shown in Fig. 2.

**Figure 3:**
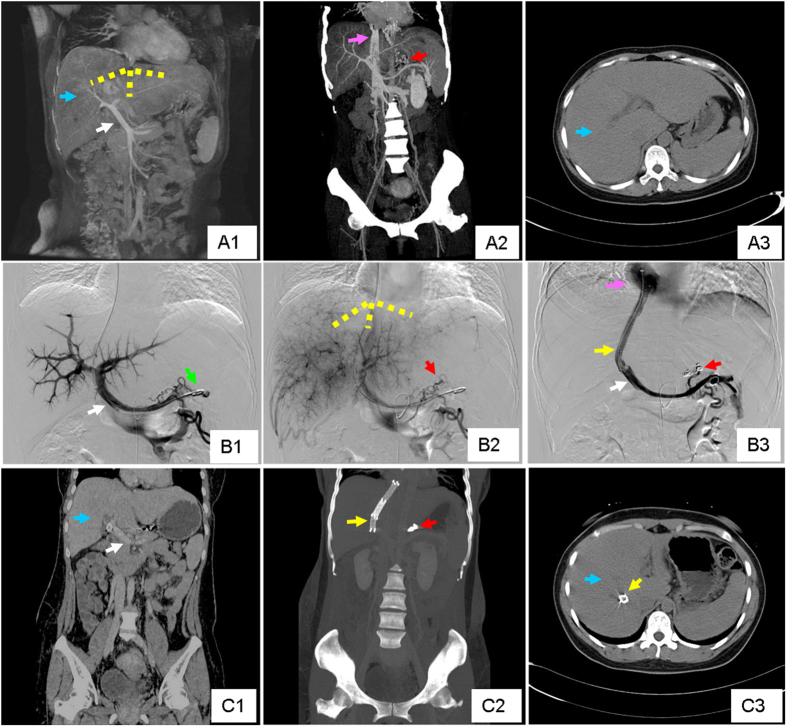
A representative case of acute BCS. This was from a 40 y/o female patient, who suffered from rapidly progressive abdominal pain, digestive discomfort and severe jaundice. (**A1,A2,A3**) Coronal image of MRPV, coronal and transverse view of CT showed diffuse occlusion of three HVs and massive liver congestion. The yellow dotted line in (**A1**) outlined the original position of occluded HVs. The Blue arrows in (**A1,A3**) highlighted the congested liver tissue. The white arrow in (**A1**) illustrated the portal vein. The pink arrow in (**A2**) emphasized the inferior vena cava and the red arrows in (**A2,B2**) pointed to the gastric esophageal varices. (**B1,B2**) Early and late phase of angiography of portal vein revealed complete occlusion of HVs. (**B3**) Angiography of portal vein after TIPS. The white arrow in (**B1**) highlighted the portal vein on angiography, and the green arrow in (**B1**) pointed to the pigtail catheter performing portal vein angiography. The yellow dotted line in (**B2**) outlined the original position of occluded HVs which couldn’t be detected. The yellow arrow pointed to the TIPS canal which linked the portal vein (white arrow) and inferior vena cava (pink arrow). The red arrow in (**B3**) pointed to the embolized gastric esophageal varices. (**C1,C2,C3**) Coronal image, 3-D reconstruction and transverse view of CT 4 weeks after TIPS demonstrated the location of the stents, and a significant decrease in liver congestion. The blue arrows in (**C1,C3**) indicated obvious relief of liver congestion. The yellow and red arrow pointed to the TIPS stents and embolized gastric esophageal varices, respectively.

**Figure 4:**
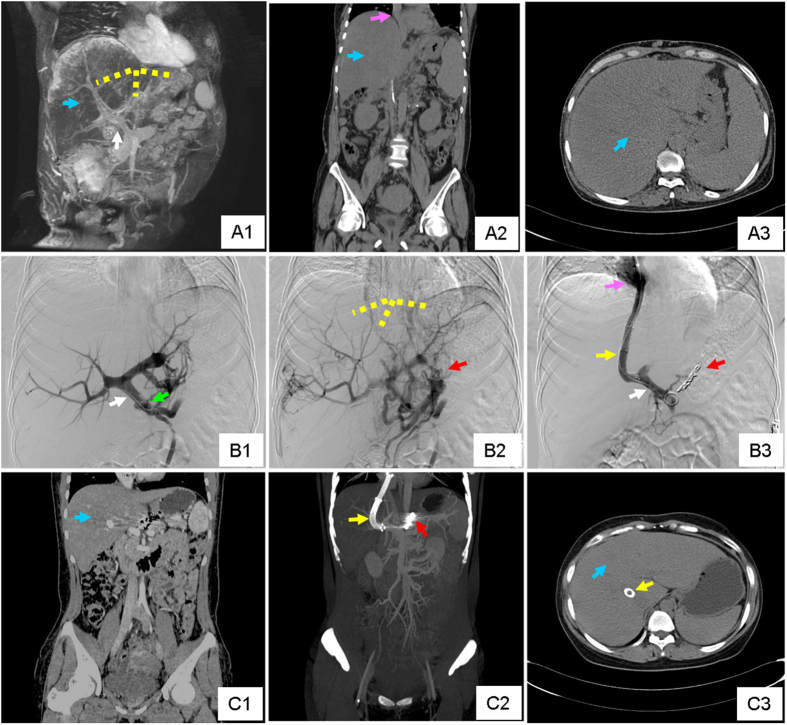
A representative case of sub-acute BCS. This was from a 27 y/o female patient, who suffered from progressive digestive discomfort and one gastrointestinal bleeding. (**A1**) Coronal image of MRPV showed diffuse occlusion of three HVs and enlargement, congestion and geographic change of the liver. (**A2,A3**). Coronal and transverse view of CT also revealed massive liver congestion. The yellow dotted line in (**A1**) outlined the original position of occluded HVs. The Blue arrow in (**A1,A2,A3**) demonstrated the congested liver tissue. The white arrow in (**A1**) illustrated the portal vein. The pink arrow in (**A2**) highlighted the inferior vena cava. (**B1,B2**) Early and late phase of angiography of portal vein revealed total occlusion of HVs and severe varicose coronary gastric vein. (**B3**) Angiography of portal vein after TIPS creation. The white arrow in (**B1**) emphasized the portal vein on angiography, and the green arrow in (**B1**) pointed to the pigtail catheter performing portal vein angiography. The yellow dotted line in (**B2**) outlined the original position of occluded HVs which couldn’t be imaged. The red arrows in (**B2**) pointed to the gastric esophageal varices. The yellow arrow pointed to the TIPS canal which linked the portal vein (white arrow) and inferior vena cava (pink arrow). The red arrow in (**B3**) pointed to the embolized gastric esophageal varices. (**C1,C2,C3**) Coronal image, 3-D reconstruction and transverse view of CT 4 weeks after TIPS showed the position of the stents, and that liver congestion had decreased obviously. The blue arrows in (**C1,C3**) indicated obvious relief of liver congestion. The yellow and red arrow pointed to the TIPS stents and embolized gastric esophageal varices, respectively.

A good clinical outcome was achieved in both groups (Figs 3, 4). Abdominal discomfort was relieved within 2 weeks after TIPS. One patient in sub-acute BCS group suffered from cervix pseudoaneurysm, and recovered after compression therapy. No other surgery-related complication was observed. The occurrence of refractory ascites was decreased in both acute and sub-acute BCS groups, and variceal bleeding was successfully prevented. TBIL level before TIPS was significantly higher in acute BCS group (203.17 ± 67.90 μmol/L) than in sub-acute group (44.82 ± 21.31 μmol/L) (P < 0.001), and efficiently decreased to a similar level to the sub-acute (29.08 ± 8.88 μmol/L vs. 27.69 ± 7.89 μmol/L) after TIPS.

Liver function was improved significantly, with ALT and AST decreased from 51.08 ± 18.46 to 17.17 ± 11.88 U/L (P < 0.001) and from 46.50 ± 19.93 to 24.33 ± 5.35 U/L (P < 0.001), respectively, in acute group, while from 58.89 ± 16.10 to 16.05 ± 9.55 U/L and from 56.04 ± 14.50 (P < 0.001) to 20.45 ± 6.46 U/L (P < 0.001), respectively, in sub-acute group.

### Follow up

All patients were followed up postoperatively for 5 years. The patients who underwent TIPS and those who underwent conservative treatments were followed separately. In TIPS groups of 91 patients, shunt dysfunction (overall 10.99%) occurred at a similar rate, i.e., 16.67% in acute BCS group (2/12) and 10.12% in sub-acute BCS group (8/79). Balloon dilation of the TIPS shunt was applied. Before the procedure, 1 out of 12 patients (8.33%) in acute BCS and 2 out of 79 patients (2.53%) in sub-acute BCS suffered from HE. During follow-up period, mild HE occurred in those patients, while 2 new patients in sub-acute BCS developed HE, and were cured after treatment. During follow-up period, 2 patients with acute BCS died (16.67%). One patient died of liver failure 1 months after TIPS, and the other died regardless to liver disease. Four patients with sub-acute BCS died (5.06%): 1 patient died of liver failure 4 months after the procedure, 1 patient die of hepatocellular carcinoma, and the other 2 patients died due to non-liver related diseases.

**Figure 5:**
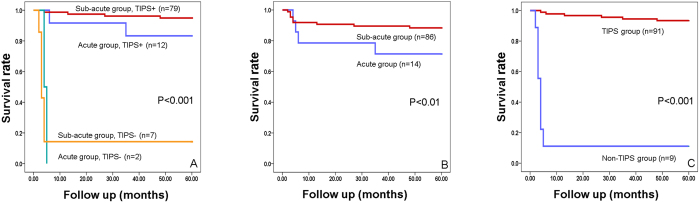
(**A**) Kaplan–Meier analysis of survival rate following BCS with diffuse occlusion of HVs. (**B**) Kaplan–Meier analysis of survival rate following BCS with diffuse occlusion of HVs in acute and sub-acute groups. (**C**) Kaplan–Meier analysis of survival rate following BCS with diffuse occlusion of HVs in TIPS and non-TIPS groups.

However, for 9 patients who didn’t undergo TIPS procedure, 2 patients with acute BCS and 6 patients out of 7 with sub-acute BCS died of liver failure within 5 months. Therefore, patients who underwent TIPS had a significantly higher survival rate than the non-TIPS treated group (Fig. 5).

## Discussion

BCS is a group of disorders resulting from obstruction of HVs, inferior vena cava (IVC) and hepatic venules. BCS affects younger to mid-aged patients with the mean age of 40 y/o^6^. Clinical symptoms of BCS vary greatly depending on the course of disease. Acute BCS is mainly caused by diffuse occlusion of HVs and presents with rapid development of abdominal pain, ascites, hepatomegaly and jaundice. The symptoms develop very fast within 6 months and always lead to liver failure and death without proper treatment. Sub-acute and chronic BCS present with progressive ascites, gastrointestinal bleeding, lower extremity edema, abdominal varices and hepatic encephalopathy. However, when diffuse occlusion of HVs occurs in sub-acute BCS, the mortality rate is also high without suitable intervention^7^.

TIPS is a procedure that can establish communication between the inflow of portal vein and the outflow of HV or IVC. TIPS has been applied for BCS since the 1990’s, and a good clinical outcome has been achieved. Qi *et al*. reviewed 160 studies on patients with BCS who underwent TIPS^8^, and found that hemodynamic and clinical improvement was significant; meanwhile, compared to liver cirrhosis, patients with BCS had lower risk of shunt dysfunction. Moreover, Tripathi D. *et al*. reported a ten-year patency rate of 72% and a survival rate of 91% after TIPS for BCS^9^. In our study, shunt dysfunction rate was as low as 10.99%, while survival rate was 83.33% and 94.94%, respectively, in acute and sub-acute BCS. The 5-year overall survival rate reached 93.41%.

Traditionally, severe jaundice with TBIL >51.3 μmol/L (3 mg/dL) was a contraindication for TIPS in end-stage liver diseases^10^. A long term study following up patients who underwent TIPS indicated that patients with MELD (Model for End-Stage Liver Disease) score >18 had a significantly higher mortality rate than those with scores of ≤ 18^11^. In addition, according to a cohort study of 220 patients undergoing TIPS, pre-TIPS bilirubin level is a powerful independent predictor of mortality of TIPS, with 1 mg/dL increase in TBIL leading to 40% increased risk of death^12^. Meanwhile, more than 90% of acute BCS cases are accompanied with severe jaundice. In our study, before TIPS, acute BCS had an average TBIL of 203.17 ± 67.90 μmol/L. However, a good clinical outcome has been achieved after TIPS. Although patients with acute BCS had a much higher TBIL level, there was no significant difference in ALT and AST level between acute and sub-acute BCS. The explanation might be, unlike liver cirrhosis, liver cell degeneration and necrosis much less occur in acute and sub-acute BCS^13^, and thus liver function can be restored after the release of hepatic congestion. In other words, severe jaundice is not a contraindication for TIPS in patients with BCS. Compared to patients who underwent conservative treatment, the much higher 5-year survival rate also supports TIPS as an efficient approach for BCS with diffuse HV occlusion despite of severe jaundice.

Although recommended for patients with BCS by American and British guidelines after diagnosis^14^,^15^, the role of anticoagulant treatment for diffuse occlusion of HVs remains controversial^16^. In our study, warfarin was given to each patient for 1 year with INR reaching 2 to 3. No anticoagulation related complication was observed, and a good shunt patency rate was achieved.

The risk of developing HE after TIPS in BCS is much lower than that in liver cirrhosis. Tripathi D. *et al*. reported that the risk of HE post-TIPS in BCS was 15%, with cirrhosis developed in 50% of patients^9^. In our study the risk of HE post-TIPS was even lower at 5.49%, and 3 out of 5 patients suffered from HE prior to TIPS. The reason of lower HE rate might be: on one hand, no cirrhosis was developed in our patient population. On the other hand, as mentioned above, although the TBIL level was extremely high in acute BCS, the liver function was well reserved. The purpose of TIPS in BCS was to decrease the PSG and reduce liver congestion. The risk of HE pre- and post-TIPS is relatively low, and HE grade is mild, therefore, HE may not be a major concern of applying TIPS to patients with BCS.

In conclusion, TIPS is an effective and safe treatment for BCS with diffuse occlusion of hepatic veins. Severe jaundice with bilirubin >3 mg/dL and pre-TIPS HE are not the contraindications for TIPS in BCS. Patients diagnosed with these features should undergo TIPS as soon as possible.

### Patients and Methods

This study has been approved by Institutional Review Board (IRB) committee at Beijing Shijitan Hospital, Capital Medical University. Informed consent was acquired from each participate before treatment. All procedures were conducted according to the guidelines approved by the ethics committee at Beijing Shijitan Hospital, Capital Medical University. Demographic and clinical information was acquired from patients who were treated in our single center from January 2007 to December 2010.

Inclusion and exclusion criteria were carefully designed to exclude the confounding factors. The inclusion criteria were: (1) Budd–Chiari syndrome with total occlusion of three hepatic veins; (2) aged between 18–70 years. The patients with one or more of the following characteristics were excluded: (1) combined with inferior vena cava and/or portal vein thrombosis; (2) combined with hemorrhage of gastrointestinal ulcer; (2) combined with malignant liver tumor or malignancies at other sites; (3) successful angioplasty of one or more HVs.

### Clinical characteristics

Before hospitalization, all patients were initially diagnosed with acute and sub-acute BCS at local hospitals. Common symptoms included abdominal discomfort, abdominal distention, severe yellow sclera and pruritus, massive ascites and other features. After administration, laboratory tests including blood routine, biochemistry and ammonia were recorded and liver function were evaluated for each patient. Ultrasound, abdominal computed tomography (CT), magnetic resonance imaging of portal vein (MRPV), gastroscopy, radiography of the upper gastrointestinal tract and inferior vena cava, as well as angiography of hepatic vein were performed on each patient. All patients were conformed with diagnosis of BCS with diffuse occlusion of all three HVs. Laboratory tests included total bilirubin (TBIL), alanine aminotransferase (ALT), aspartate aminotransferase (AST) and blood ammonia. Clinical features including abdomen pain, ascites, variceal bleeding, abdominal and chest varices, hepatic encephalopathy (HE) and gastric esophageal varices were documented. Anti-coagulation with warfarin was administered to each patient after TIPS and low molecular weight heparin was applied before the international normalized ratio (INR) reached 2–3.

Patients were classified into acute BCS and sub-acute BCS groups. The patients with a course of disease of no more than 4 weeks were defined as acute BCS, while patients with a course of disease of more than 6 weeks and less than 24 weeks were defined as sub-acute BCS. Operative and postoperative parameters of patients in two groups were compared.

### TIPS procedure

TIPS was suggested to each patient. The technique has been described previously^3^. TIPS was performed under local anesthesia in the Interventional Radiology Suite of Beijing Shijitan Hospital, Capital Medical University. Right jugular venous access was gained with a 10 F sheath of Rösch-Uchida Transjugular Liver Access Set (Cook). A 5 F multipurpose catheter was inserted into the hepatic vein. Angiography was performed and diffuse occlusion of the hepatic venules was identified. A puncture needle was advanced into the portal vein through the liver parenchyma from inferior vena cava. Then the guide wire was placed into the portal vein through the 10 F sheath. Portal vein angiography was performed with a 5-F pigtail catheter. The portal vein pressure and right atrium pressure was measured to calculate the portosystemic pressure gradient (PSG). The varicose coronary gastric vein was embolized to prevent potential bleeding. TIPS shunt was dilated with an angioplasty balloon of 8 or 10 mm diameter, and then a covered stent with a diameter of 8 or 10 mm was deployed. If the stent was not long enough, one additional stent with the same diameter was applied to extend the shunt. PSG was calculated again after the shunt had been established.

### Postoperative observation and treatments

The patients were under close monitoring during perioperative period. Low molecular weight heparin (5000 IU, twice per day) was subcutaneously injected for 5 days, and then warfarin was given for 1 year. Coagulation function of each patient was examined every 15 days to ensure the INR reaching 2 to 3. Intravenous injection of branched chain amino acid and oral administration of lactulose were also applied to prevent HE. Complications, including abdominal cavity hemorrhage, HE and hepatic failure were observed postoperatively.

During this period, patients who were refused to be treated with TIPS received conservative treatment. Anti-coagulation, branched chain amino acids and oral administration of lactulose were also applied to these patients. Overall survival time was compared between acute BCS group (TIPS+, TIPS−) and sub-acute BCS group (TIPS+, TIPS−) as well as between TIPS group and non-TIPS group.

### Follow up

The patients were followed up at 3- and 6-months after the procedures, and re-examinations at every 6 months up to 5 years. Clinical information was recorded, and color ultrasonography, abdominal CT and esophagography were repeated at each time-point. So did laboratory investigations, including TBIL, ALT, AST and ammonia.

### Statistical analysis

SPSS for Windows version 17.0 was used for statistical analysis, with paired-sample t test for comparisons and χ^2^ test for qualitative data. Numerical data was summarized as frequencies, and continuous variables was shown as mean ± standard deviation (SD). Data for survival were analyzed using the Kaplan–Meier method and log-rank test. A p value of < 0.05 was considered to be statistically significant.

## Additional Information

**How to cite this article**: He, F. *et al*. Transjugular intrahepatic portosystemic shunt for Budd–Chiari syndrome with diffuse occlusion of hepatic veins. *Sci. Rep.* **6**, 36380; doi: 10.1038/srep36380 (2016).

**Publisher’s note:** Springer Nature remains neutral with regard to jurisdictional claims in published maps and institutional affiliations.

## References

[b1] Potthoff A. & Bahr M. J. Response to Time to resize the role of liver transplant for Budd-Chiari syndrome. Liver Int 35, 2339 (2015). 25818650 10.1111/liv.12837

[b2] Cheng D. . Comparative study of MRI manifestations of acute and chronic Budd-Chiari syndrome. Abdom Imaging 40, 76–84 (2015). 25063237 10.1007/s00261-014-0193-y

[b3] He F. L. . Transjugular intrahepatic portosystemic shunt for severe jaundice in patients with acute Budd-Chiari syndrome. World J Gastroenterol 21, 2413–2418 (2015). 25741149 10.3748/wjg.v21.i8.2413 PMC4342918

[b4] Mancuso A. TIPS for Budd-Chiari syndrome: time to anticipate treatment. Liver Int 34, 325 (2014). 24650135 10.1111/liv.12544

[b5] Goel R. M., Johnston E. L., Patel K. V. & Wong T. Budd-Chiari syndrome: investigation, treatment and outcomes. Postgrad Med J 91, 692–697 (2015). 26494427 10.1136/postgradmedj-2015-133402

[b6] Rosenqvist K. . Endovascular treatment of symptomatic Budd-Chiari syndrome - in favour of early transjugular intrahepatic portosystemic shunt. Eur J Gastroenterol Hepatol 28, 656–660 (2016). 26958788 10.1097/MEG.0000000000000621

[b7] Qi X., Yang M., Fan D. & Han G. Transjugular intrahepatic portosystemic shunt in the treatment of Budd-Chiari syndrome: a critical review of literatures. Scand J Gastroenterol 48, 771–784 (2013). 23506234 10.3109/00365521.2013.777775

[b8] Tripathi D. . Good clinical outcomes following transjugular intrahepatic portosystemic stent-shunts in Budd-Chiari syndrome. Aliment Pharmacol Ther 39, 864–872 (2014). 24611957 10.1111/apt.12668

[b9] Rössle M. TIPS: 25 years later. J Hepatol 59, 1081–1093 (2013). 23811307 10.1016/j.jhep.2013.06.014

[b10] Schepke M. . Comparison of MELD, Child-Pugh, and Emory model for the prediction of survival in patients undergoing transjugular intrahepatic portosystemic shunting. Am J Gastroenterol 98, 1167–1174 (2003). 12809844 10.1111/j.1572-0241.2003.07515.x

[b11] Rajan D. K., Haskal Z. J. & Clark T. W. Serum bilirubin and early mortality after transjugular intrahepatic portosystemic shunts: results of a multivariate analysis. J Vasc Interv Radiol 13, 155–161 (2002). 11830621 10.1016/s1051-0443(07)61932-0

[b12] Aydinli M. & Bayraktar Y. Budd-Chiari syndrome: etiology, pathogenesis and diagnosis. World J Gastroenterol 13, 2693–2696 (2007). 17569137 10.3748/wjg.v13.i19.2693 PMC4147117

[b13] DeLeve L. D. . Vascular disorders of the liver. Hepatology 49, 1729–1764 (2009). 19399912 10.1002/hep.22772 PMC6697263

[b14] Watson H. . Guidelines on the diagnosis and management of heparin-induced thrombocytopenia: second edition. Br J Haematol 159, 528–540 (2012). 23043677 10.1111/bjh.12059

[b15] Plessier A., Rautou P. E. & Valla D. C. Management of hepatic vascular diseases. J Hepatol 56, 25–38 (2012). 10.1016/S0168-8278(12)60004-X 22300463

[b16] Fitsiori K. . Transjugular intrahepatic portosystemic shunt for the treatment of Budd-Chiari syndrome patients: results from a single center. Cardiovasc Intervent Radiol 37, 691–697 (2014). 23860938 10.1007/s00270-013-0697-9

